# Differential processing of dissolved and particulate organic matter by deep-sea sponges and their microbial symbionts

**DOI:** 10.1038/s41598-020-74670-0

**Published:** 2020-10-15

**Authors:** Martijn C. Bart, Anna de Kluijver, Sean Hoetjes, Samira Absalah, Benjamin Mueller, Ellen Kenchington, Hans Tore Rapp, Jasper M. de Goeij

**Affiliations:** 1 Department of Freshwater and Marine Ecology, Institute for Biodiversity and Ecosystem Dynamics, University of Amsterdam, PO Box 94248, 1090 GE Amsterdam, The Netherlands; 2 Department of Earth Sciences, Utrecht University, Utrecht, The Netherlands; 3 Department of Fisheries and Oceans, Bedford Institute of Oceanography, Dartmouth, NS Canada; 4 Department of Biological Sciences, University of Bergen, Bergen, Norway

**Keywords:** Ecophysiology, Coral reefs, Element cycles, Symbiosis

## Abstract

Deep-sea sponges create hotspots of biodiversity and biological activity in the otherwise barren deep-sea. However, it remains elusive how sponge hosts and their microbial symbionts acquire and process food in these food-limited environments. Therefore, we traced the processing (i.e. assimilation and respiration) of ^13^C- and ^15^N-enriched dissolved organic matter (DOM) and bacteria by three dominant North Atlantic deep-sea sponges: the high microbial abundance (HMA) demosponge *Geodia barretti*, the low microbial abundance (LMA) demosponge *Hymedesmia paupertas*, and the LMA hexactinellid *Vazella pourtalesii*. We also assessed the assimilation of both food sources into sponge- and bacteria-specific phospholipid-derived fatty acid (PLFA) biomarkers. All sponges were capable of assimilating DOM as well as bacteria. However, processing of the two food sources differed considerably between the tested species: the DOM assimilation-to-respiration efficiency was highest for the HMA sponge, yet uptake rates were 4–5 times lower compared to LMA sponges. In contrast, bacteria were assimilated most efficiently and at the highest rate by the hexactinellid compared to the demosponges. Our results indicate that phylogeny and functional traits (e.g., abundance of microbial symbionts, morphology) influence food preferences and diet composition of sponges, which further helps to understand their role as key ecosystem engineers of deep-sea habitats.

## Introduction

Towards the end of the nineteenth century, ship-based dredging expeditions of the HMS *Porcupine*, *Lightning*, and *Challenger* started to reveal the occurrence of sponges at water depths up to a 1000 m^1,2^. Since then, many sponge species have been found to be abundant in various deep-sea ecosystems, such as cold-water coral reefs and sponge grounds^3–5^. In the North-Atlantic Ocean, massive ball-shaped demosponges from the *Geodiidae* family form large aggregations known as “osturs” or “cheese bottoms”^6^, while thin encrusting species occur in high diversity and abundance on the Rockall Bank west of Scotland and the Porcupine Bank west of Ireland^7^. Hexactinellids form glass sponge grounds, either monospecific or as part of a more diverse community, such as the Schulz Bank on the Mid-Atlantic Ridge^8^ and the *Vazella pourtalesii* grounds on the Scotian Shelf^9,10^. Despite the fact that the deep-sea is generally considered to be barren and food-limited, these deep-sea sponge ecosystems are known to be hotspots of biodiversity and organic carbon cycling^10–12^.

Nevertheless, the ecological role of sponges in the deep-sea is still relatively undescribed, although an increasing body of literature is starting to reveal their importance. They promote biodiversity by increasing habitat complexity^10,13,14^ and provide foraging and nursery grounds for mobile taxa, such as fish^15,16^. Moreover, deep-sea sponges are important in benthic-pelagic coupling of resources^17,18^. However, while deep-sea ecosystems are increasingly threatened by anthropogenic impacts, such as oil and gas exploration^19^, mining^20,21^, and fisheries^22,23^, many questions concerning the physiology and ecological functioning of sponges are still unanswered. A particularly relevant question is how the deep-sea sponge holobiont, i.e. the animal host and its numerous and diverse community of associated microorganisms^24,25^, takes up, processes, and releases nutrients in the seemingly food-deprived deep-sea environment.

In general, sponges are efficient filter-feeders that utilize a wide variety of particulate food sources ranging from bacterio- and phytoplankton to detrital particles, and even zooplankton^18,26,27^. Moreover, in recent years it has become increasingly evident that many sponges, including dominant North Atlantic deep-sea species^28,29^, use dissolved organic matter (DOM) as main food source^30^. DOM constitutes the largest pool of reduced carbon in the ocean, and is therefore the largest potential marine organic food source^31^. However, DOM appears to represent only a minor fraction of the food intake of most invertebrates^32^, and is generally considered to be recycled through bacterial processing^33^. Consequently, when various shallow water sponge holobionts were found to feed predominantly—often 70 to > 90% of their daily carbon intake—on DOM^34–36^, this capacity was attributed to their microbial symbionts^37–39^. Sponges with high abundances of associated microbes (HMA; species-specific microbial communities with up to four orders of magnitude higher bacterial concentrations than the surrounding sea water) were suggested to be better equipped to utilize DOM, than those with low microbial abundances (LMA; community composition and abundances of microbes similar to surrounding sea water)^39–41^. However, recent evidence from complementary studies using different approaches suggests that microbial abundance may not determine the capacity of sponges to feed on DOM, since many LMA sponges were found to take up DOM^29,35,36,42^ and some even at higher uptake rates than HMA sponges^28,43,44^. Furthermore, DOM has been shown to be assimilated by both sponge host and bacterial symbiont cells^42,44^, ultimately visualized at a (sub)cellular level^45,46^. However, the initial uptake and possible translocation between host and symbionts has yet to be established.

The capability to utilize dissolved food sources might be especially relevant in highly food-deprived environments, such as the deep-sea, where food availability is considered to primarily dependent on a vertical flux of sinking particular organic matter and local bacterioplankton production^47,48^. Indeed, first evidence of four dominant North-Atlantic sponge species, including one massive vase-shaped LMA hexactinellid, one encrusting sheet-shaped LMA and two massive ball-shaped HMA demosponges, showed that none of these species could acquire sufficient carbon from particulate organic matter (POM) food sources alone to meet their respiratory demand. Instead, all tested species relied on DOM to balance their metabolic requirements^28^. However, no information is yet available on the role of the animal host versus its symbionts in the processing of particulate and dissolved organic food sources, which hampers our understanding of different strategies these deep-sea sponges may have to acquire food.

We therefore studied how deep-sea sponges from different phylogenetic classes, and with high and low abundances of microbial symbionts process dissolved and particulate food sources. We traced the assimilation and respiration of two ^13^C- and ^15^N-enriched food sources (DOM and bacteria) by three different (i.e. based on phylogeny, abundances of associated microbes, morphology) dominant North Atlantic deep-sea sponge species—*Geodia barretti* (Demospongiae, HMA, massive), *Vazella pourtalesii* (Hexactinellidae, LMA, massive), and *Hymedesmia paupertas* (Demospongiae, LMA, encrusting)—using ex situ incubations. Subsequently, phospholipid-derived fatty acid (PLFA) biomarker analysis was performed to elucidate the role of the sponge host and the associated microbes in the utilization of the two isotope-tracer food sources. The use of PLFA biomarkers in combination with isotope tracer experiments has been shown to successfully provide insight in differential processing of isotope-tracer food sources by sponge host cells and bacterial symbionts in various studies e.g., Refs.^41,42,44^.

## Results

### Bulk tracer processing of dissolved organic matter (DOM) and bacteria

**Figure 1:**
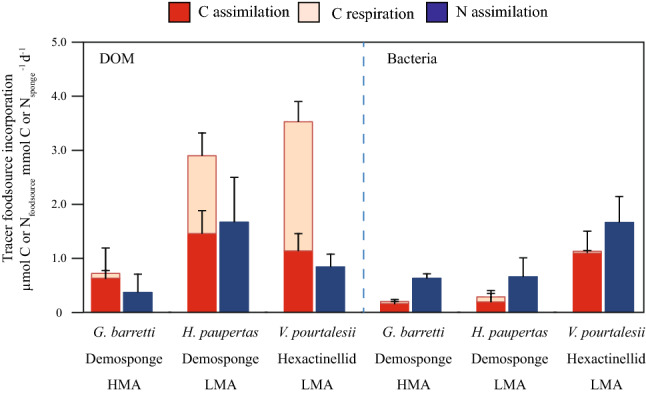
Processing of tracer (^13^C- and ^15^N-) DOM (left) and bacteria (right) by deep-sea sponges. Dark red bars represent tracer carbon (C_DOM_ or C_bac_) assimilation rates, light red bars represent tracer carbon respiration rates and blue bars represent tracer nitrogen (N_DOM_ or N_bac_) assimilation rates in µmol C or N_food source_ mmol C or ${\text{N}}_{{{\text{sponge}}}}^{ - 1}$ d−1. *HMA* high microbial abundance sponge species, *LMA* low microbial abundance sponge species.

Both the algal-derived dissolved and bacterial-derived particulate organic food sources were assimilated and respired by all three sponge species (Fig. 1).

#### DOM processing rates and assimilation efficiency

**Figure 2:**
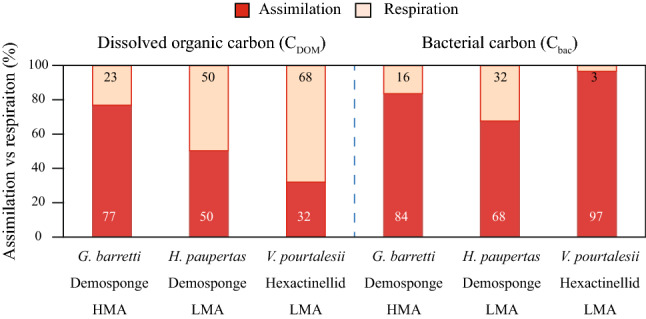
Relative assimilation and respiration of ^13^C-tracer DOM (left) and bacteria (right). Dark red bars represent relative tracer carbon (C_DOM_ or C_bac_) assimilation, light red bars represent relative tracer carbon respiration, as % of total processing of each food source per sponge species tested. *HMA* high microbial abundance sponge species, *LMA* low microbial abundance sponge species.

The high microbial abundance (HMA) sponge *Geodia barretti* showed significantly lower average uptake rates of carbon from the dissolved food source (C_DOM_) (0.7 ± 0.6 µmol C_DOM_ mmol $${\text{C}}_{{{\text{sponge}}}}^{ - 1}$$ d^−1^; mean ± SD throughout text unless stated otherwise) than the low microbial abundance (LMA) species *Hymedesmia paupertas* and *Vazella pourtalesii* (2.9 ± 0.8 and 3.5 ± 0.7 µmol C_DOM_ mmol $${\text{C}}_{{{\text{sponge}}}}^{ - 1}$$ d^−1^, respectively) (Fig. 1, Table 1). These differences were mainly caused by the order of magnitude lower C_DOM_ respiration rates of the HMA species (0.09 ± 0.05 µmol C_DOM_ mmol $${\text{C}}_{{{\text{sponge}}}}^{ - 1}$$ d^−1^) compared with the two LMA species *H. paupertas* and *V. pourtalesii*, respectively (4.7 ± 0.8 and 1.4 ± 0.4 µmol C_DOM_ mmol $${\text{C}}_{{{\text{sponge}}}}^{ - 1}$$ d^−1^, Fig. 1). No significant differences were found in nitrogen (N_DOM_) uptake between *G. barretti* (0.4 ± 0.3 µmol N_DOM_ mmol $${\text{N}}_{{{\text{sponge}}}}^{ - 1}$$ d^−1^), *H. paupertas* (1.7 ± 0.8 µmol N_DOM_ mmol $${\text{N}}_{{{\text{s
ponge}}}}^{ - 1}$$ d^−1^), and *V. pourtalesii* (0.8 ± 0.2 µmol N_DOM_ mmol $${\text{N}}_{{{\text{sponge}}}}^{ - 1}$$ d^−1^) (Table 1). Despite possessing the lowest processing rates, the HMA sponge *G. barretti* showed a higher assimilation-to-respiration efficiency (77%) compared to *H. paupertas* and *V. pourtalesii* (50 and 32%, respectively), although only the difference between *G. barretti* and *V. pourtalesii* was significant (Fig. 2, Table 2).

**Table 1:** Results of the pairwise comparisons for two-factor Monte Carlo PERMANOVAs testing for differences in total dissolved organic carbon and nitrogen (C_DOM_ and N_DOM_) and bacterial carbon and nitrogen (C_bac_ and N_bac_) processing rates between the deep-sea sponge species *Geodia barretti* (Demosponge, HMA), *Hymedesmia paupertas* (Demosponge, LMA) and *Vazella pourtalesii* (Hexactinellid, LMA)*.*

	df	*t*	*p*_(MC)_	Unique Permutations
**C**_**DOM**_** processing**				
G. barretti * V. pourtalesii	4	5.249	**0.007**	10
G. barretti * H. paupertas	4	3.611	**0.022**	10
V. pourtalesii * H. paupertas	4	0.979	0.376	10
**C**_**bac**_** processing**				
G. barretti * V. pourtalesii	5	4.573	**0.008**	35
G. barretti * H. paupertas	5	0.535	0.621	35
V. pourtalesii * H. paupertas	4	2.762	**0.052**	10
**N**_**DOM**_** processing**				
G. barretti * V. pourtalesii	4	1.980	0.119	10
G. barretti * H. paupertas	4	2.512	0.062	10
V. pourtalesii * H. paupertas	4	1.662	0.181	7
**N**_**bac**_** processing**				
G. barretti * V. pourtalesii	5	4.430	**0.006**	35
G. barretti * H. paupertas	5	0.390	0.714	35
V. pourtalesii * H. paupertas	4	2.963	**0.037**	10

df = degrees of freedom, *t* = *t*-statistic, *p*_(MC)_ = Monte Carlo *p* value. Values in bold are statistically significant (*p* < 0.05).

**Table 2:** Results of the pairwise comparisons for the two-factor Monte Carlo PERMANOVAs testing for differences between the deep-sea sponge species *Geodia barretti* (Demosponge, HMA), *Hymedesmia paupertas* (Demosponge, LMA) and *Vazella pourtalesii* (Hexactinellid, LMA), in the assimilation-to-respiration efficiencies of dissolved organic carbon (C_DOM_) and bacterial carbon (C_bac_).

	df	*t*	*p*_(MC)_	Unique Permutations
**C**_**DOM**_				
G. barretti * V. pourtalesii	4	3.718	**0.019**	10
G. barretti * H. paupertas	4	2.221	0.089	10
V. pourtalesii * H. paupertas	4	11.314	**0.001**	4
**C**_**bac**_				
G. barretti * V. pourtalesii	5	1.969	0.105	35
G. barretti * H. paupertas	5	2.410	0.060	15
V. pourtalesii * H. paupertas	4	273.020	**< 0.001**	4

df = degrees of freedom, *t* = *t*-statistic, *p*_(MC)_ = Monte Carlo *p* value. Values in bold are statistically significant (*p* < 0.05).

#### Bacteria processing rates and assimilation efficiency

For the bacterial food source, significant differences in processing rates were found between the hexactinellid *V. pourtalesii*, showing the highest total bacterial C and N processing rates (1.1 ± 0.4 and 1.6 ± 0.5 µmol C and N_bac_ mmol C and ${\text{N}}_{{{\text{sponge}}}}^{ - 1}$ d^−1^), and the two demosponges *H. paupertas* (0.3 ± 0.3 and 0.7 ± 0.3 µmol C and N_bac_ mmol C and ${\text{N}}_{{{\text{sponge}}}}^{ - 1}$ d^−1^) and *G. barretti* (0.2 ± 0.1 and 0.6 ± 0.1 µmol C and N_bac_ mmol C and ${\text{N}}_{{{\text{sponge}}}}^{ - 1}$ d^−1^) (Fig. 1, Table 1). Respiration rates were low, 0.04 ± 0.04, 0.09 ± 0.11 and 0.07 ± 0.02 µmol C_bac_ mmol ${\text{C}}_{{{\text{sponge}}}}^{ - 1}$ d^−1^ for *G. barretti*, *H. paupertas* and *V. pourtalesii*, respectively, and comparable between the three species. The hexactinellid *V. pourtalesii* also showed the highest assimilation-to-respiration efficiency for the bacterial food source (97%), compared with demosponges *H. paupertas* and *G. barretti* (68 and 84%), although this efficiency was only significantly different between *V. pourtalesii* and *H. paupertas* (Fig. 2, Table 2).

#### C:N ratios of assimilation of DOM and bacterial food source

Averaged over all sponge species, C:N ratios for the assimilation of the DOM food source were significantly higher than for the bacterial food source (1.2 ± 0.3 versus 0.4 ± 0.2, respectively (*t* = 7.0, *df* = 17, *p* < 0.0001)).

### Sponge host and symbiont tracer processing of DOM and bacterial food source

#### PLFA profiles of deep-sea sponges and food substrates

**Figure 3:**
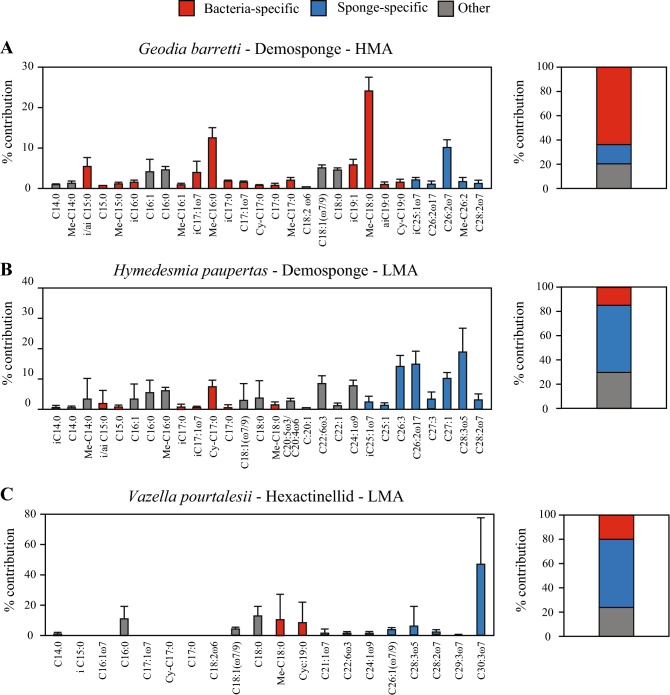
Phospholipid-derived fatty acid (PLFA) profiles and total percentages of bacteria-specific, sponge-specific, and unspecific PLFAs of deep-sea sponges. Bacteria-specific (red), sponge-specific (blue), and unspecific PLFAs (grey) are shown for (**A**) *Geodia barretti (n* = 11), (**B**) *Hymedesmia paupertas* (*n* = 7), and (**C**) *Vazella pourtalesii* (*n* = 6). Values are presented as mean (± SD) percentages of the total PLFA content. Stacked bars depict the total relative contributions of bacteria-specific (red), sponge-specific (blue), and unspecific (grey) PLFAs for each species. *HMA* high microbial abundance, *LMA* low microbial abundance.

The three investigated sponge species possess distinct PLFA profiles (Fig. 3). Total PLFA carbon was on average 0.7 ± 0.6% (*V. pourtalesii*, *n* = 6), 0.9 ± 0.4% (*G. barretti*, *n* = 11) and 1.9 ± 0.6% (*H. paupertas*, *n* = 7) of the total sponge holobiont carbon biomass. The HMA sponge *G. barretti* showed the highest relative contribution of bacteria-specific PLFAs (62.7 ± 3.0%; depicted in red in Fig. 3), which is significantly higher than LMA sponges *H. paupertas* (8.6 ± 6.9%) and *V. pourtalesii* (17.7 ± 23.9%), respectively (Table S4). In contrast, the proportions of sponge-specific PLFAs were at least four times higher in the LMA species *H. paupertas* and *V. pourtalesii* (68.7 ± 11.1% and 58.0 ± 52.1%, respectively) compared to the HMA sponge *G. barretti* (16.4 ± 3.0%) (Table SS4. The sponge-specific PLFA biomarkers (depicted in blue in Fig. 3) of the demosponges *H. paupertas* and *G. barretti* contained typical very-long chained demosponge PLFAs, such as C26:2, C28:2, C26:3 and C28:3. Sponge-specific PLFAs of the LMA hexactinellid sponge *V. pourtalesii* were dominated by one very long-chained PLFA (C30:3ω7). A considerable part of the PLFAs of all three species could not be assigned exclusively to either bacterial or sponge origin (20.9–27.2%) and are depicted in grey in Fig. 3. An overview of the relative abundance of each PLFA per species is presented in Supplementary Table S3 online.

PLFA profiles of both food sources: two algal-derived DOM types (cyanobacteria-DOM fed to *V. pourtalesii*, and diatom-DOM fed to *G. barretti* and *H. paupertas*) and bacteria, are shown in supplementary Fig. S3 and Table S3. DOM obtained from the lyophilized cyanobacterium *Agmenellum quadruplicatum* contained mainly C16:0 (38.4%) and C16:1 (36.9%). The diatom-derived DOM also contained mainly C16:1 (64.8%), C16:0 (12.7%), and the typical diatom biomarker C20:5ω3 (10.2%). The bacterial culture only contained C16:0 (22.4%), C18:1 (59.9%) and C18:0 (17.7%) in detectable amounts.

#### Assimilation of DOM and bacterial food source in host and symbionts

**Figure 4:**
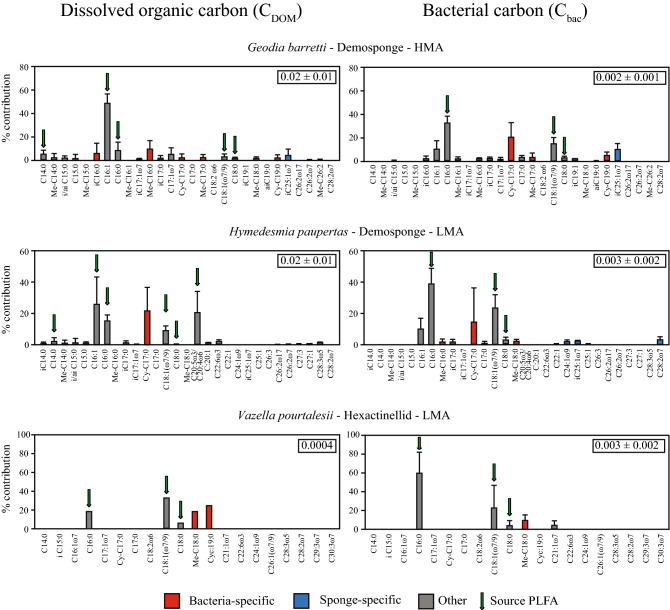
Incorporation of DO^13^C and bacterial ^13^C tracer into sponge-specific, bacteria-specific, and unspecific phospholipid-derived fatty acids (PLFAs). Values are presented as percentages of total C_DOM_ and C_bac_ tracer incorporated into each individual PLFA (mean ± SD). Bacteria-specific (red), sponge-specific (blue), and unspecific PLFAs (grey) are shown for (**A**) *Geodia barretti* (DOM: *n* = 3, bacteria: *n* = 4), (**B**) *Hymedesmia paupertas* (DOM: *n* = 3, bacteria: *n* = 3), and (**C**) *Vazella pourtalesii* (DOM: *n* = 1, bacteria: *n* = 3). Green arrows indicate the presence of the fatty acid in the DOM or bacterial source fed to the sponge. *HMA* high microbial abundance, *LMA* low microbial abundance. Total PLFA incorporation rate is presented in the box in the upper right corner of each panel in µmol CDOM or ${\text{C}}_{{{\text{sponge}}}}^{ - 1}$ d^−1^ (mean ± SD).

The stable isotope-enriched tracer DOM and bacterial food source were clearly assimilated into PLFAs of all three deep-sea sponge holobionts (Fig. 4). Demosponges *G. barretti* (HMA) and *H. paupertas* (LMA)*,* showed assimilation rates of DOM into total PLFAs of one order of magnitude higher (0.02 ± 0.01 and 0.02 µmol ± 0.01 C_DOM_ mmol ${\text{C}}_{{{\text{sponge}}}}^{ - 1}$ d^−1^, respectively) than for bacteria (0.002 ± 0.001 and 0.003 ± 0.002 µmol C_bac_ mmol ${\text{C}}_{{{\text{sponge}}}}^{ - 1}$ d^−1^, respectively). In contrast, the opposite was found in the hexactinellid LMA *V. pourtalesii*, with one order of magnitude lower assimilation rates of DOM into PLFAs (0.0004 µmol C_DOM_ mmol ${\text{C}}_{{{\text{sponge}}}}^{ - 1}$ d^−1^) compared to the bacterial source (0.003 ± 0.002 µmol C_bac_ mmol ${\text{C}}_{{{\text{sponge}}}}^{ - 1}$ d^−1^) (Fig. 4). ). For DOM-fed sponges, the majority of labelled PLFAs found in *G. barretti* (62.9%), for *H. paupertas* (66.9%) and for *V. pourtalesii* (55.0%) originated from PLFAs present in the food sources (e.g., C14:0, C16:0, C16:1, C18:0, C18:1, C20:5ω3, depending on the source, indicated with the arrows in Fig. 4). For the bacterial food source, the dominant fatty acids C16:0, C18:1 and C18:0 accounted for 24.5% (*G. barretti*), 34.8% (*H. paupertas*) and 89.3% (*V. pourtalesii*) of the total tracer PLFA incorporation. This indicates that the major fatty acids of both food sources were directly ingested by the sponges. Additionally, both food sources were traced in PLFAs that were not originally present in the food source, indicating de novo synthesis of these PLFAs by the sponge holobionts. Most of these newly synthesized PLFAs are either bacteria-specific (i/aiC15:0, iC17:0, Cy-C17, Cy-C19, Me-C16:0, Me-C18:0) or non-specific (C16:1) in all sponge species and irrespective of food source (8–45% of total tracer carbon assimilation depicted in red, Fig. 4, Supplementary Fig. S4 online). Sponge-specific PLFAs only contributed marginally to the newly synthesized PLFA fraction (0–8% depicted in blue, Fig. 4, Fig. S4), irrespective of food source and sponge species. However, a large part of the labelled PLFAs were neither bacterial- nor sponge specific (54–91% depicted in grey, Fig. 4, Supplementary Fig. S4 online), and could thus be de novo synthesized by either the host or its symbionts. Interestingly, bacteria-fed sponge specimens showed a higher percentage of labelled sponge-specific PLFAs compared to DOC-feed specimens in all tested sponge species (*G. barretti*: 4.5 vs. 8.8%, *H. paupertas*: 2.8 vs. 6.0%, *V. pourtalesii*: 0.0 vs. 0.2%). However, the combined effect of species and food sources was not statistically significant (df = 2, *F* = 0.359, *p*_*perm*_ = 0.78).

## Discussion

Here, we show the processing (i.e. assimilation and respiration) of a dissolved (DOM) and a particulate (bacteria) food source for a selection of three dominant deep-sea sponges from two different phylogenetic classes that differ in functional traits, such as the abundance of microbial symbionts and morphology. Our results corroborate recent and increasing evidence^28,30,49^ that DOM-processing is not restricted to high microbial (HMA) sponges as is commonly suggested^40,41,50^, but that low microbial abundance (LMA) sponges are capable of processing DOM at even higher rates than HMA species. However, the HMA sponge in this study, *Geodia barretti*, did show the highest assimilation-to-respiration efficiency of DOM, i.e. relatively more food is put into new biomass instead of lost through respiration. Overall, assimilation-to-respiration efficiencies were lower for DOM than for bacteria. For the particulate food source (bacteria), not bacterial abundance (i.e. HMA or LMA), but phylogenetic class distinguished its processing rates, with highest rates found in the hexactinellid compared to the two demosponges. The hexactinellid *Vazella pourtalesii* also showed a very high assimilation-to-respiration efficiency (97%) and higher processing rates into compound-specific phospholipid fatty acids (PLFAs) for bacterial food compared with DOM, whereas the demosponges *G. barretti* and *Hymedesmia paupertas* showed the opposite. Both tracer food sources were, within the time frame of the incubation experiments, foremost assimilated into shorter-chained microbial symbiont specific PLFAs by all sponges, and not in the very-long-chained sponge host-specific PLFAs. However, this does not quantify symbiont and host processing, since the majority of DOM and bacterial food tracers were found in non-host/symbiont-specific PLFAs. Based on the observations made here on a limited number of tested species, we cannot draw conclusions on the exact drivers (e.g., phylogeny, abundance and composition of microbiome, morphology) in the processing of dissolved and particulate food sources. To fully understand how phylogenetic and anatomical differences affect food processing by deep-sea sponges, more studies on a broader spectrum of sponge types are needed.

### Bulk processing of DOM and bacteria as food source

It is commonly assumed that sponges with high microbial abundances are better equipped to take up dissolved food than LMA sponges^40,41,50^. However, DOC processing rates (i.e. the sum of assimilation and respiration) for the LMA species studied here (*H. paupertas* and *V. pourtalesii*) are four to five times higher than for the HMA sponge *G. barretti*, mainly due to higher respiration of the DOM source. Overall, assimilation rates (0.6–1.4 µmol C_DOM_ mmol C_sponge_ d^−1^, 0.4–1.7 µmol N_DOM_ mmol N_sponge_ d^−1^) are in the same range as the only reported rates of (deep-sea coral-derived) DOM processing for the deep-sea LMA sponge *Hymedesmia coriacea* (1.7 ± 1.6 µmol C_DOM_ mmol C_sponge_ d^−1^, 2.0 ± 2.0 µmol N_DOM_ mmol N_sponge_ d^−1^^29^. Thus, despite differences in microbial abundances or DOM sources (i.e. diatom-, cyanobacterial-, coral-derived DOM), DOM-tracer assimilation rates seem to be comparable among deep-sea sponges.

The assimilation-to-respiration efficiencies indicate that DOM is assimilated most efficiently by the HMA sponge *G. barretti*, even though at lower rates. The complex interaction between sponge host and its abundant community of microbial symbionts might result in an uncoupling between uptake, assimilation, and respiration, leading to the very low respiration rates of DOM found for *G. barretti*. For example, respired C from the DOM source may be fixed by microbial symbionts into organic matter via chemoautotrophy. In fact, Van Duyl et al.^51,52^ showed that sponge holobionts are capable of fixing inorganic C. Additionally, *G. barretti* is known to possesses multiple associated bacteria phyla that support CO_2_ fixation, such as *Nitrospinae* and *Chloroflexi*^53–55^.

In all sponges, assimilation-to-respiration efficiencies appeared to be highest for the bacterial food source. Especially the hexactinellid *V. pourtalesii* showed a stunning 97% assimilation-to-respiration efficiency. This corroborates earlier findings that sponges, and hexactinellids in particular, are very efficient in filter-feeding and assimilating (tracer) bacteria^18,42^. Still, DOM potentially constitutes a much larger proportion of their daily diet than bacteria^28^, mainly due to the order of magnitude higher ambient concentration of DOM-derived C and N in seawater^35^. The different assimilation-to-respiration efficiencies of DOM versus bacteria suggest that food sources may serve different purposes for sponge nutrition as was previously hypothesized by Refs.^28,56^. This is further corroborated by the significantly lower C:N ratios of bacterial assimilation compared with the assimilation of DOM for all species, which indicates that sponges differentially process C and N from these sources, i.e. process relative more N from particulate over dissolved food sources. Preferential assimilation of bacterial N has also been found for the cold-water sponge *Spongosorites coralliophaga*^56^. Considering the high dissolved inorganic nitrogen (DIN) efflux rates found in many sponges from both tropical and deep-sea habitats^54,57,58^, preferential assimilation of N might facilitate the sponge’s maintenance of stoichiometric homeostasis. Additionally, bacteria contain high fractions of essential constituents, such as amino acids, fatty acids, and vitamins^59^, which are essential building blocks for anabolic processes. In contrast, algal-derived DOM, such as the here used diatom- and cyanobacteria-DOM, contains a relatively high fraction of neutral sugars^60,61^, which can be rapidly respired. Glucose, for example, was found to be almost exclusively respired by sponges^35,56^. Therefore, we hypothesize that DOM may serve as the main energy source for deep-sea sponges to sustain their minimal energetic requirements, while supplementation with bacteria and other high-quality particulate food sources is essential to support anabolic processes (e.g., somatic growth, reproduction, and cell turnover), particularly during episodic food pulses after phytoplankton blooms. This also explains why sponges can be found in areas with locally enhanced particle supply, even where this supply of particulate organic matter (POM) alone is not enough to sustain their minimal respiratory demands^62^.

It is important to mention that both bacteria and DOM used as food sources in this study were laboratory-made. This could affect our results as bacteria grown in culture may have different C- and N-contents compared to bacteria naturally occurring in seawater^63^. Furthermore, the composition and bioavailability of freshly produced, artificial DOM (lysed diatom/cyanobacterial cells) may differ from the marine DOM pool, particularly in the deep-sea, where DOM is presumed to be largely refractory^44,64^. Nevertheless, all deep-sea sponges used in this study proved capable of utilizing dissolved food sources, irrespective of the abundance of associated microbes. Yet, the processing of DOM within sponge holobionts differed between species.

### Processing of dissolved and particulate food sources into host and symbiont PLFAs

Our results on the assimilation of labelled DOM and bacteria into sponge host and symbiont PLFAs suggests that within sponge holobionts, DOM is primarily assimilated by bacterial symbionts, which corroborates with earlier findings by Rix and colleagues^29,44^. However, we cannot be conclusive here for three reasons: (1) A large portion of DOM assimilated into PLFAs (54–91%) could not be assigned to either host- or symbiont-specific biomarkers. (2) Assimilated DOM may have been incorporated into sponge cells, but not (yet) metabolized into PLFAs. For deep-sea sponges, it might simply take longer than the 24–48 h duration of the incubations to synthesize long-chained, sponge-specific PLFAs from shorter precursor PLFAs^65^. A recent study by Achlatis et al.^46^ visualized at a subcellular level that sponge cells, in contrast to bacterial cells, first store carbon in other cellular components or in other lipids than PLFAs. (3) Sponge-mediated assimilation may have been underestimated, since part of the newly synthesized sponge-specific PLFAs may have been already lost through cell turnover. Evidence is accumulating in tropical sponges that choanocytes are the dominant sponge cells that process DOM^45,46^ and these cells may be partly lost as detrital waste through shedding^41,45,66^. Cell loss was not monitored here, due to the methodological complexity, yet should be incorporated into future (tracer) metabolic studies on deep-sea sponges.

De novo synthesis of PLFAs confirms the active processing of labelled food sources, rather than the simple re-use of source PLFAs. For example, the labelled bacteria-specific PLFAs Cy-C17:0, Me-C16:0 and Me-C18:0 were not present in the bacterial food source, but could only be found in the sponge-holobiont at the end of the incubation, thus indicating de novo synthesis by sponge-associated bacteria. In the same incubations, also labelled sponge-specific PLFAs (e.g., C22:1ω7, C24:1ω9, i-C25:1ω7, C25:1, C28:2ω7) were found in all tested deep-sea sponge species after the incubations. Similarly, incubations with labelled DOM as food source resulted in the synthesis of labelled bacteria-specific (e.g., i/aiC15:0, i-C17:0, Me-C16:0, Cy-C17:0, Me-C18:0) and sponge-specific PLFAs (*G. barretti*: i-C25:1ω7, C26:2ω7, Me-C26:2ω7; *H. paupertas*: C26:2ω7, C27:3, C27:1, C28:3ω7, C28:2ω7), that were not present in the DOM substrate provided. This confirms that while ^13^C from bacteria is more efficiently assimilated into PLFAs by the sponge holobiont, also DOM can serve as source material for the de novo synthesis of bacterial- and sponge-specific PLFAs. Thus, both associated bacteria and the sponge host are involved in the processing of particulate (i.e. bacteria) and dissolved food (i.e. DOM).

## Conclusion

Deep-sea sponges are capable of assimilating C and N from both dissolved and particulate food sources, but differentially process the two types of food. Contrary to the conventional view, the LMA sponges tested here, *H. paupertas* and *V. pourtalesii* processed DOM at higher rates than the HMA sponge *G. barretti*, but at lower assimilation-to-respiration efficiencies. For bacteria, the highest assimilation-to-respiration efficiency and processing rates were found in the hexactinellid LMA species *V. pourtalesii*. This sponge also showed a higher incorporation rate of bacteria over DOM into PLFAs, opposite of the rates found for the demosponges *G. barretti* and *H. paupertas.* We hypothesize that, in general, bacteria are more efficiently assimilated and serve as an N source to support anabolic processes of deep-sea sponges, while DOM primarily serves as energy source to sustain maintenance metabolism of the sponge holobiont. Our results further indicate that the phylogenetic class and functional traits, such as abundance of microbial symbionts, of sponges influence their food preferences and diet composition, which further helps to understand the role of sponges as key ecosystem engineers of the deep-sea.

## Materials and methods

### Study areas, sponge collection, and maintenance

This study used the following deep-sea sponge species: *Vazella pourtalesii* (Hexactinellidae, LMA)*, Geodia barretti* (Demospongiae, HMA) and *Hymedesmia paupertas* (Demospongiae, LMA) (Fig. S1, Supplementary Table S1 online). All sponges were analysed by transmission electron microscopy to determine species and LMA/HMA status [Refs.^58,67^, Hans-Tore Rapp personal observations, data not shown]. Sponges were collected by remotely-operated vehicle (ROV) during two research cruises in 2016 and 2018. Whole *V. pourtalesii* individuals were collected attached to their rocky substrate at ~ 300 m water depth, during the Hudson cruise 2016–2019 (September 2016) at the Emerald Basin (43°59′49.0″N 62°46′15.7″W), an area with a large monospecific *V. pourtalesii* population^68^. Sponges were kept in the dark in a 1000-L holding tank and transported without air exposure to the Bedford Institute of Oceanography, Dartmouth, Nova Scotia, Canada. In the lab, sponges were kept in the dark in a 1000-L flow-through holding tank, through which sand-filtered seawater from the Bedford Basin was continuously pumped at 7 L h^−1^. A chiller was used to maintain water temperature at 8 °C. Whole *G. barretti and H. paupertas* individuals were collected attached to their rocky substrate during the G.O. Sars cruise 2018108 (August 2018) in the western Barents Sea (70°47′13.9″N 18°03′23.8″E), an area dominated by large *G. barretti* individuals^69^. These sponges were kept on board the research vessel in the dark in 20-L flow-through tanks in a climate room at 6 °C. Seawater was directly pumped in from a water depth of 6 m at 30 L h^−1^. All (^13^C- and ^15^N-) tracer DOM incubations with *H. paupertas* and *G. barretti* were performed on board the ship and tracer bacteria incubations were performed at the University of Bergen, Norway. Sponges were transported without air exposure. In Bergen, sponges were kept in a dark climate room (8 °C) in multiple 20-L flow-through aquarium systems. Flow originated from unfiltered water pumped from 200 m water depth from the outer fjord near Bergen at ~ 50 L h^−1^ with a temperature ranging from 6–8 °C.

### Preparation of ^13^C- and ^15^N-labelled food sources

^13^C- and ^15^N-enriched dissolved (algal-derived) and a particulate (i.e. bacterial) organic food source tracer were administered to the three sponge species: individuals of each species received either the dissolved organic (DOM) or the bacterial food source (*see* below for details on replication) A detailed culture and extraction protocol for both food sources can be found in Supplementary Materials and Methods online. For *V. pourtalesii*, tracer DOM was extracted from 1 g ^13^C- and ^15^N-labelled lyophilized algal cells (*Agmenellum quadruplicatum*) (Cambridge isotopes CNLM-455-1, Eurisotop). For *G. barretti* and *H. paupertas*, tracer DOM was extracted from axenic diatom (*Phaeodactylym tricornicum*) cultures on F/2 medium^70^ amended with 80% ^15^N-NaNO_3_ (Cambridge isotopes NLM-157, Eurisotop) and 100% ^13^C-NaHCO_3_ (Cambridge isotopes CLM-441, Eurisotop). Different algal-derived DOM-sources were used because in 2016, time constraints did unfortunately not allow us to prepare diatom algal batch cultures before the experiments with *V. pourtalesii* were performed, therefore DOM was extracted from pre-labelled algae, directly obtained from Cambridge isotopes. Both DOM-sources were obtained by lysing the algal cells and filtering the lysate over a 0.2 µm polycarbonate filter. The filtrate was collected, lyophilized, and analysed by EA-IRMS for C and N content and isotopic composition. Before adding the DOM to the incubations, aliquots of 5 mL were made by dissolving the lyophilized DOM in MilliQ. Tracer bacteria were pre-labelled with ^13^C and ^15^N according to de Goeij et al.^42^. In short, prefiltered seawater containing natural bacterial communities was concentrated and added to M63 medium^71^. As C source, 1 g L^−1 13^C-glucose (glucose D U-13C6 99%, Cambridge isotopes CLM-1396, Eurisotop) was added and (NH_4_)_2_SO_4_ in the original recipe was replaced by 1.2 g L^−1 15^N- NH_4_Cl as N source (99% ^15^N, Cambridge isotopes NLM-467-5, Eurisotop). Labelled bacteria were concentrated and resuspended in 0.2 µm filtered seawater before dividing in aliquots and storing at 4 °C.

### Sponge incubations with ^13^C- and ^15^N-labelled food sources

All sponges were allowed to acclimatize for a minimum of 1 week prior to the incubation experiments^72^. All individuals appeared healthy during their time in the aquaria, and throughout the experiments. All oscula were open and active pumping was confirmed using fluorescent dye. None of the used sponges showed signs of tissue necrosis and no mortality occurred through the experiments. Individual sponges were enclosed in acid-washed (0.4 mol L^−1^ HCl) flow chambers with magnetic stirring devices^43^. During the experiments, chambers were kept in the dark and in a water bath to maintain a constant seawater temperature during the incubations (ranging from 6 to 9 °C depending on the incubation). Chambers were closed without trapping air in the system. During incubations, oxygen was continuously measured with an OXY-4 mini oxygen sensor (PreSens). Oxygen profiles of the sponge incubations are depicted in Supplementary Fig. S2 online.

Incubation time was based on the consumption of oxygen within the incubation chamber and the biomass of the sponge (Supplementary Tables S1, S2, and Supplementary Fig. S2, online). Different individuals were used for each food source, and each sponge received multiple pulses of labelled food sources to ensure detectable enrichment in host and symbionts. Each *V. pourtalesii* specimen (*n* = 3 for DOM, *n* = 3 for bacteria) was incubated for 2 × 24 h. *H. paupertas* (*n* = 3 for DOM, *n* = 3 for bacteria) and *G. barretti* (*n* = 3 for DOM, *n* = 4 for bacteria) individuals were incubated for 3 × 8 h. Labelled substrates were added with sterile syringes. DOM was added to a final concentration of 80 µmol L^−1^ dissolved organic carbon (DOC) for all three tested sponge species (Supplementary Table S2 online). Bacteria were added to a final concentration of 1 × 10^6^ labelled bacteria mL^−1^ for *V. pourtalesii* (approximately 16 µmol L^−1^ bacterial carbon (BC)) and 0.5 × 10^6^ labelled bacteria mL^−1^ for *G. barretti* and *H. paupertas* (approximately 12 µmol L^−1^ BC). In between subsequent incubations, water was replaced with non-labelled fresh seawater and a new pulse of tracer substrate (in aforementioned concentrations) was added. Seawater incubations without sponges (*n* = 3 for DOM, *n* = 3 for bacteria for incubations in 2017 and 2018, respectively) were performed accordingly to serve as controls.

### Assimilation and respiration of labelled food sources

After the incubations, all sponges were thoroughly rinsed with 0.2 µm filtered seawater to ensure no labelled residue adhered to the outside of the sponge, and dipped in MilliQ to remove salts. Specimens were then dried (48 h at 60 °C) and dry weight (DW) determined. Then, sponge tissue was homogenized with mortar and pestle and stored in a desiccator until further analysis. Samples for organic carbon content analysis were decalcified with 4 mol L^−1^ HCl to remove inorganic carbon and lyophilized (24 h). Approximately 10 mg per sample (in silver capsules) was analysed on an Elemental Analyser (Elementar Isotope cube) coupled to an isotope ratio mass spectrometer (BioVision) for simultaneous measurement of organic C and total N content as well as ^13^C:^12^C and ^15^N:^14^N ratios.

To quantify respiration of the labelled food sources, duplicate water samples for dissolved inorganic ^13^carbon (DI^13^C) were taken with acid-washed polycarbonate syringes, directly after adding the labelled substrate (t_0_) and at the end of each incubation (t_end_) (δ^13^DIC). Samples were transferred through a 0.2 µm polycarbonate syringe filter into 3 mL exetainers without trapping air in the vial, and poisoned with 5 µL supersaturated HgCl. Samples were stored at 4 °C until further analysis. 20 mL air-tight glass vials were filled with 3 drops of 90% H_3_PO_4_ and flushed with helium for 5 min, after which 0.7 mL of sample was added. Na_2_CO_3_ (2 and 10 mmol L^−1^) and Li_2_CO_3_ (2 mmol L^−1^) served as reference standards for concentration and isotope signature. Samples were analysed by gas chromatograph-isotope ratio mass spectrometry (GC-IRMS) on a Thermo GasBench-II coupled to a Delta-V advantage. As references, ^13^C-Na_2_CO_3_ and Li_2_CO_3_ (LSVEC) were calibrated versus NBS-19 and NBS-18 on a Kiel-MAT253. Standard methods according to manual of the manufacturer were used. The standard deviation of the measurements was < 0.05 ‰.

To assess the assimilation and respiration of tracer food sources, ^13^C:^12^C and ^15^N:^14^N ratios, are expressed in standard delta notation as:

$$\delta^{13} C {\text{or }}\delta^{15} N \left( \permil \right) = \left( { \frac{{R_{sample} }}{{R_{ref - 1} }} } \right) \times 1000$$

where R is the ratio of ^13^C:^12^C or ^15^N:^14^N in the sample (R_sample_; i.e. sponge tissue) relative to reference material: Vienna Pee Dee Belemnite for C (R_ref_ = 0.01118) and atmospheric nitrogen for N (R_ref_ = 0.00368). Total bulk enrichment was calculated as the excess fractional abundance of ^13^C or ^15^N (E) in the samples (F_sample_) compared with their background [i.e. the natural content of ^13^C or ^15^N in the samples before adding tracer (F_background_)]:

$$E_{sample} = F_{sample} - F_{background}$$

where F is the fractional abundance of heavy isotope (^13^C or ^15^N) calculated as:

$$F_{sample\;or\;background} = \frac{{\delta^{13} C }}{{\delta^{13} C + \delta^{12} C }} \;or\; \frac{{\delta^{15} N }}{{\delta^{15} N + \delta^{14} N }}$$

and

$$F_{sample\;or\;background} = \left( {\frac{{\delta^{13} C \;{\text{or}}\;\delta ^{15} N }}{1000 + 1}} \right) \times R_{ref}$$

Total assimilation and respiration rates for C and assimilation rates for N were calculated by multiplying the excess fractional abundance (E_sample_) by the total C_org_ or N_org_ content (μmol) of the tissue.

### Phospholipid-derived fatty acid extraction and data analysis

Phospholipid-derived fatty acids (PLFAs) were analysed for bacteria-fed (*G. barretti*: *n* = 4, *V. pourtalesii*: *n* = 3, and *H. paupertas*: *n* = 3) as well as DOM-fed sponge specimens (*G. barretti*: *n* = 3, *V. pourtalesii*: *n* = 1 (unfortunately, there was only sufficient tissue to perform PLFA analysis for one individual), and *H. paupertas*: *n* = 3), and compared to non-labelled backgrounds of the respective sponges (*V. pourtalesii*: *n* = 2, *G. barretti*: *n* = 3, *H. paupertas*: *n* = 2). PLFAs were extracted according to a modified Bligh and Dyer Extraction protocol^73,74^ (see Ref.^75^ and Supplementary Materials and Methods online for detailed protocol).

Concentration and isotopic composition of individual PLFAs was determined with a gas chromatograph-combustion-interface isotope ratio mass spectrometer (GC-c-IRMS) consisting of a HP G1530 GC (Hewlett-Packard) connected to a delta-plus IRMS via a type-III combustion interface (Thermo Finnigan, Bremen) on an analytical non-polar column (CP-sil 5, 25 m × 0.32 mm × 0.12 µm). Fatty acid identification was carried out with GC mass spectrometry (MS) (Finnigan Trace GC) using the same column and settings as for IRMS. Results were compared with pre-existing datasets from Utrecht University based on equivalent chain length (ECL).

Data-analysis was performed using the R-package “Rlims”^76^. Concentration of PLFA’s within the sample were calculated based on the peak areas of the respective PLFA (*A*_*PLFA*_), the peak area of the standard C19:0 (*A*_*19:0*_), and carbon amount of the standard C19:0 (*C*_*19:0*_):

$$C_{PLFA} = \frac{{A_{PLFA} \div A_{19:0} \times C_{19:0} }}{gs \times f} \times \frac{n}{n + 1}$$

where *gs* is the total amount of the sample (mg), *f* is the fraction of DCM recovered during the extraction, and *n* is the number of C-atoms in the PLFA. The last factor is to correct for the methyl group that was added during the analytic procedure.

Stable isotope ratios (^13^C) of individual fatty acids were calculated from FAME data by correcting for the carbon atom in the methyl group that was added during derivatization. Carbon isotope ratios were calculated relative to Vienna Pee Dee Belemnite (VPDB) and tracer carbon incorporation was quantified through determining above background ^13^C content in the extracted phospholipids.

$$\delta^{13} C_{PLFA} = \frac{{\left( {n + 1} \right)\delta^{13} C_{PLFA} - \delta^{13} C_{methanol} }}{n }$$

where *n* is the number of carbon atoms in a fatty acid. PLFAs are described as CX:YωZ, where X is the number of C-atoms in the PLFA, Y is the number of double bonds, and Z is the position of the first double bond counted from the methyl (ω) end of the molecule. Prefix “Me” indicates mid-methyl branching, prefix “i” (iso) and “ai” (anteiso) indicate a methyl group one or two carbon away from the methyl end, respectively. Prefix “Cy” indicates presence of a cyclo-ring.

### Statistical analysis

All statistical analyses were conducted in PRIMER-E version 6^77^ with the PERMANOVA + add-on^77^. PERMANOVAs with Monte Carlo tests were performed as this method is robust for small sample sizes and when assumptions of normality and homogeneity are not met^78^. Individual two-factor PERMANOVAs based on Euclidian distance, with type 3 (partial) sum of squares and unrestricted permutation of raw data (9999 permutations) were used to test for differences in total processing rates of DOM- and bacterial-derived C and N between and within species and assimilation-to-respiration efficiencies for all species per carbon source. Post hoc pairwise comparisons were carried out when species or food source were identified as a significant factor (Tables 1 and 2). To compare pooled average assimilation C:N ratios between DOM and bacteria, a Welch’s *t*-test was performed. Normality was visually checked with a Q–Q plot. To test for differences between species in the contribution of bacteria-specific and sponge-specific PLFAs to the total PLFA profile of each species, A one factor PERMANOVA based on Euclidian distance, with type 3 (partial) sum of squares and unrestricted permutation of raw data (9999 permutations) was used (Supplementary Table S4 online).

## Supplementary information

Supplementary Information.

## Data Availability

All datasets will be made publicly available upon publication through the meta-data record PANGAEA.
