# Supplementary material for: Differential processing of dissolved and particulate organic matter by deep-sea sponges and their microbial symbionts
Source: Sci Rep. 2020 Oct 15;10:17515. doi: 10.1038/s41598-020-74670-0 (PMC7567089; doi:10.1038/s41598-020-74670-0)

**Supplementary materials & methods**

**Differential processing of dissolved and particulate organic matter by deep-sea sponges and their microbial symbionts**

**Authors:**

Martijn C. Bart^1*^, Anna de Kluijver^2^, Sean Hoetjes^1^, Samira Absalah^1^, Benjamin Mueller^1^, Ellen Kenchington^3^, Hans Tore Rapp^4^ Jasper M. de Goeij^1^

^1^Department of Freshwater and Marine Ecology, Institute for Biodiversity and Ecosystem Dynamics, University of Amsterdam, PO Box 94248, 1090 GE Amsterdam, the Netherlands

^2^ Department of Earth Sciences, Utrecht University, Utrecht, the Netherlands

^3^Department of Fisheries and Oceans (DFO), Bedford Institute of Oceanography, Dartmouth, Nova Scotia, Canada

^4^Department of Biological Sciences, University of Bergen, Bergen, Norway

*corresponding author, E-mail: m.c.bart@uva.nl

**Preparation of ^13^C- and ^15^N-labeled food sources**

We administered a ^13^carbon (C)- and ^15^nitrogen (N)-enriched particulate (i.e. bacterial) and dissolved (algal-derived) organic food source tracer to the three sponge species: Individuals of each species received either the DOM or the bacterial food source (*see* below for details on replication). For *V. pourtalesi*, tracer DOM was extracted from 1 g ^13^C- and ^15^N-labeled lyophilized algal cells (*Agmenellum quadruplicatum* cyanobacterium, > 98 % atom purity, Cambridge Isotope Laboratories CNLM-455-1, Eurisotop). Briefly, the cell powder was resuspended in ultrapure water and sonicated for 15–20 min, followed by 10 min centrifugation at 750 x *g*. The supernatant was then filtered over a 0.7 µm GF/F filter and subsequently over a 0.2 µm polycarbonate membrane filter. The resulting DOM filtrate (< 0.2 µm) was lyophilized and analyzed by elemental analyser–isotope ratio mass spectrometry (EA-IRMS) for C and N content and isotopic composition. For *G. barretti* and *H. paupertas*, tracer-DOM was prepared by culturing axenic diatom *Phaeodactylym tricornicum* in multiple 2-L Fernbach flasks on F/2 medium (Guillard 1975) amended with 80 % ^15^N-NaNO_3_ (Cambridge Isotope Laboratories CLM-157, Eurisotop) and 100 % ^13^C-NaHCO_3_ (Cambridge Isotope Laboratories CLM-441, Eurisotop). Non-labeled axenic *P. tricornicum* pre-cultures were added (60 mL) to 1 L of sterile labeled F/2 medium in a flow cabinet. Diatoms were grown at 20 °C on a 12:12 light:dark cycle. After 10 d, diatoms were concentrated on a 0.45 µm filter (147 mm diameter) and carefully removed from the filter by flushing with sterile artificial seawater. Subsequently, the collected diatoms were centrifuged for 10 min at 750 x *g*, the supernatant was removed, and the pellet was frozen at -20 °C. To lyse the cells and release the ^13^C- and ^15^N-labeled DOM, frozen diatoms were lyophilized in a FD5515 *Ilchin Biobase* freeze drier, after which ultrapure water was added and the solution was placed in an ultrasonic bath for 10 min. Lastly, DOM solution was filtered over a 0.7 µm GF/F filter and subsequently over a 0.2 µm polycarbonate filter. The filtrate was collected, lyophilized, and analyzed by EA-IRMS for C and N content and isotopic composition. Before adding the DOM to the incubations, aliquots of 5 mL were made by dissolving the lyophilized DOM in MilliQ.

Tracer bacteria were pre-labeled with ^13^C and ^15^N according to de Goeij et al. [41]. In short, bacteria were concentrated by prefiltering seawater containing natural bacterial communities over 0.7 μm; GF/F and subsequent ultrafiltration (0.2 µm; Vivaflow). The inoculum was added to M63 medium [68] amended with thiamine (0.00001 %) and MgSO_4_ (67 µmol L^-1^). As C source, 1 g L^-1^ ^13^C-glucose (glucose D U-13C6 99 %, Cambridge Isotope Laboratories CLM-1396, Eurisotop) was added and (NH_4_)_2_SO_4_ in the original recipe was replaced by 1.2 g L^-1^ ^15^N- NH_4_Cl as N source (99 % ^15^N, Cambridge Isotope Laboratories NLM-467-5, Eurisotop). The culture was grown for 48 h in the dark at 25 ˚C and labeled bacteria were concentrated by centrifuging (5 min, 10.000 x *g*), rinsing the pellet in 0.2 µm filtered seawater, centrifuging again, and resuspending the pellet in 0.2 µm filtered seawater before dividing in aliquots and storing at 4 °C.

**Phospholipid-derived fatty acid extraction**

For each extraction, 50 mg of ground and lyophilized sponge tissue was weighed in a glass centrifuge tube. To each sample, 31 mL Bligh and Dyer Extraction mix (Dichloromethane (DCM, Pestinorm ≥ 99.6 %) : Methanol, (MeOH, Pestinorm ≥ 99.7 %) : Phosphate buffer (8.7 g K_2_HPO_4_ L milliQ^-1^, pH 8) mixed in the ratio 2 : 1 : 0.8) was added. Subsequently, samples were vortexed, placed in an ultrasonic bath for 10 min, and placed on a shaker for at least 2 h. Hereafter, another 8 mL DCM and Phosphate-buffer was added, after which the samples were vortexed and centrifuged for 3 min at 100 x *g* at room temperature. Samples were placed at -20 °C overnight, or until two phases (the DCM phase containing the lipids and the water phase containing other organic molecules) were visually separated. The bottom DCM phase was collected and evaporated to dryness in a Turbovap LV (Biotage) at 40 °C under a stream of N_2_- gas (max 5 psi pressure). Samples were dissolved in 2 mL DCM : MeOH (1 : 1), of which 1 mL was used for column fractionation.

The total lipids fraction was separated in different polarity classes using a 10 mL glass pipette with extracted cotton and 2 g activated silica gel. Samples were dissolved in DCM (0.5 mL) and added to the column. Subsequently, DCM (7 mL) was added to remove the glycolipid fraction, acetone (7 mL) was added to collect the neutral lipid fraction. Lastly, 15 mL of methanol was added to collect the phospholipid fraction. Methanol was subsequently evaporated in a Turbovap LV (Biotage) at 40 °C under a stream of N_2_- gas (maximum of 5 psi pressure).

PLFAs were derivatized using mild alkaline methylation to yield fatty acid methyl esters (FAMEs). To each sample, 1 mL 0.2 mol L^-1^ methanolic NaOH and 1 mL methanol : toluene mix (1 : 1 v/v) were added, together with 40 µg C19:0 FAME (≥ 99.5 %, Sigma Aldrich, USA) (i.e. according to the nomenclature explained in the last paragraph of the methods) as internal standard. Samples were incubated for 15 min at 37 °C, after which 2 mL hexane, 0.3 mL 1 mol L^-1^ acetic acid and 2 mL MiliQ were added. Samples were vortexed and the (top) hexane phase was collected and evaporated. Lastly, samples were diluted in 10-50 uL hexane with 4 µg C12:0 FAME (≥ 99.5 %, Sigma Aldrich, USA) added as second internal standard. All samples were stored at -20 °C until analysis.

**Supplementary tables & figures**

**Supplementary Table S1. Overview of sponge biomass and natural isotopic ratios for all deep-sea sponge specimens incubated.** HMA Average background delta values for ^13^C and ^15^N are presented for deep-sea sponge species *Geodia barretti* (Demosponge, HMA), *Hymedesmia paupertas* (Demosponge, LMA) and *Vazella pourtalesii* (Hexactinellid, LMA). Carbon (C) and nitrogen (N) content are presented as % of dry weight (DW). Isotopic delta values for ^13^C and ^15^N, after incubations with ^13^C- and ^15^N-isotope-enriched dissolved organic matter (DOM) and bacteria (BAC), are presented for all specimens incubated.

| **Sponge** | **Phylogenetic class** | **HMA/LMA** | **Background**  **δ^13^C** | **Background**  **δ^15^N** | **Sponge individual**  **#** | **Dry weight (g)** | **C content**  **(% DW)** | **N content**  **(% DW)** | **Tracer food source** | **Δ^13^C** | **Δ^15N^** |
| --- | --- | --- | --- | --- | --- | --- | --- | --- | --- | --- | --- |
| Geodia barretti | Demospongiae | HMA | -18.5 | 11.5 |  |  |  |  |  |  |  |
|  |  |  |  |  | 1 | 26.5 | 17.6 | 4.82 | DOM | -16.7 | 17.4 |
|  |  |  |  |  | 2 | 17.8 | 22.9 | 6.58 | DOM | 37.5 | 200.1 |
|  |  |  |  |  | 3 | 41.2 | 21.7 | 5.99 | DOM | 16.7 | 114.2 |
|  |  |  |  |  | 1 | 15.4 | 17.8 | 4.77 | BAC | -12.7 | 144.6 |
|  |  |  |  |  | 2 | 19.9 | 13.8 | 3.73 | BAC | -10.1 | 171.4 |
|  |  |  |  |  | 3 | 24.1 | 15.8 | 4.13 | BAC | -10.3 | 206.9 |
|  |  |  |  |  | 11 | 6.1 | 12.5 | 3.28 | BAC | -12.8 | 164.5 |
| Hymedesmia paupertas | Demospongiae | LMA | -17.1 | 16.7 |  |  |  |  |  |  |  |
|  |  |  |  |  | 1 | 0.99 | 14.53 | 3.52 | DOM | 56.6 | 309.6 |
|  |  |  |  |  | 2 | 1.31 | 12.40 | 1.98 | DOM | 74.0 | 718.5 |
|  |  |  |  |  | 3 | 0.66 | 12.48 | 2.09 | DOM | 32.5 | 364.2 |
|  |  |  |  |  | 1 | 0.090 | 11.75 | 3.13 | BAC | 2.2 | 275.8 |
|  |  |  |  |  | 3 | 0.41 | 11.46 | 2.69 | BAC | -13.2 | 206.0 |
| Vazella pourtalesii | Hexactinellida | LMA | -20.2 | 19.0 |  |  |  |  |  |  |  |
|  |  |  |  |  | 2 | 1.64 | 4.40 | 0.96 | DOM | 117.0 | 353.5 |
|  |  |  |  |  | 4 | 1.06 | 5.28 | 1.17 | DOM | 224.4 | 605.1 |
|  |  |  |  |  | 5 | 1.25 | 5.23 | 1.17 | DOM | 171.2 | 455.2 |
|  |  |  |  |  | 3 | 2.93 | 5.10 | 1.22 | BAC | 223.2 | 1098.5 |
|  |  |  |  |  | 7 | 3.54 | 7.06 | 1.55 | BAC | 193.2 | 1016.5 |
|  |  |  |  |  | 8 | 7.61 | 6.53 | 1.42 | BAC | 90.4 | 614.7 |

**Supplementary Table S2. Incubation durations, food source concentration and percentage labeling of ^13^C and ^15^N.** Added concentrations of dissolved organic carbon (DOC) and nitrogen (DON) are based on incubation time, amount of food pulses, incubation volume, and the percentage of C and N present in the substrate as measured by IRMS.

|  | | **Incubation time (h)** | **Incubation volume (L)** | **DOC added (****µmol L^-1^)** | **DON**  **added (µmol L^-1^)** | **Bacterial C added (µmol L^-1^)** | **Bacterial N added (µmol L^-1^)** | **Atm % ^13^C** | **Atm % ^15^N** |
| --- | --- | --- | --- | --- | --- | --- | --- | --- | --- |
|  | **^13^C – ^15^N DOM incubations** | | | | | | | | |
| Geodia barretti | | 3 x 8 | 3 x 3 | 80 | 15 |  |  | 54 | 69 |
| Hymedesmia paupertas | | 3 x 8 | 3 x 6 | 80 | 15 |  |  | 54 | 69 |
| Vazella pourtalesii | | 2 x 24 | 2 x 2 | 80 | 7 |  |  | 99 | 93 |
|  | **^13^C – ^15^N Bacteria incubations** | | | | | | | | |
| Geodia barretti | | 3 x 8 | 3 x 3 |  |  | 12 | 1 | 47 | 99 |
| Hymedesmia paupertas | | 3 x 8 | 3 x 2 |  |  | 12 | 1 | 47 | 99 |
| Vazella pourtalesii | | 2 x 24 | 2 x 6 |  |  | 16 | 7 | 95 | 99 |

**Supplementary Table S3.** Relative abundance of each PFLA to the total PLFA content of each deep-sea sponge species and substrate. Values are in percentages of total PLFAS.

| **ECL** | **UIPAC notation** | **Omega notation** | **Biomarker** | ***Geodia barretti*** | ***Hymedesmia paupertas*** | ***Vazella pourtalesii*** | ***Diatom DOM*** | ***Cyano. DOM*** | ***Bacterial culture*** |
| --- | --- | --- | --- | --- | --- | --- | --- | --- | --- |
|  |  |  |  | *n* = 11 | *n* = 7 | *n* = 6 | *n* = 1 | *n* = 1 | *n* = 1 |
| 13.51 | C13:0 (12-CH_3_) | iC14:0 | Bacteria | - | 0.33 ± 0.57 |  |  |  |  |
| 13.78 | C14:0 | C14:0 | - | 0.96 ± 0.18 | 0.22 ± 0.37 | 0.27 ± 0.48 | 3.98 | 10.54 |  |
| 14.34 | C14:0 (?-CH_3_) | Me-C14:0 | Bacteria | 1.41 ± 0.27 | 3.01 ± 7.25 |  |  |  |  |
| 14.58 | C14:0 (12-CH_3_/13-CH_3_) | *ai/i-*C15:0 | Bacteria | 5.59 ± 1.18 | 1.70 ± 2.91 | 0.05 ± 0.12 |  |  |  |
| 14.89 | C15:0 | C15:0 | Bacteria | 0.55 ± 0.11 | 0.24 ± 0.42 |  |  |  |  |
| 15.03 | C15:0 (?-CH_3_) | Me-C15:0 | Bacteria | 1.32 ± 0.40 |  |  |  |  |  |
| 15.42 | C15:0 (13-CH_3_) | iC16:0 | Bacteria | 1.52 ± 0.60 |  |  |  |  |  |
| 15.77 | C16:1 (11/9/5) | C16:1 (ω5/7/9) | - | 4.20 ± 2.57 | 3.00 ± 3.64 | 0.19 ± 0.43 | 64.76 | 38.37 | 22.44 |
| 16.00 | C16:0 | C16:0 | - | 4.51 ± 0.63 | 3.86 ± 3.33 | 8.09 ± 5.04 | 12.73 | 36.88 |  |
| 16.21 | C16:1 (12-CH_3_) | Me-C16:1 | Bacteria | 0.80 ± 0.33 |  |  |  |  |  |
| 16.34 |  | iC17:1ω7 | Bacteria | 3.85 ± 2.01 |  |  |  |  |  |
| 16.46 | C16:0 (8,9,10) | 8/9/10-Me-C16:0 | Bacteria | 12.54 ± 1.97 | 0.35 ± 0.93 |  |  |  |  |
| 16.64 | C16:0 (15-CH_3_) | *i*-C17:0 | Bacteria | 1.86 ± 0.31 | 0.79 ± 1.13 |  |  |  |  |
| 16.72 | C17:1 (10Z) | C17.1ω7 | Bacteria | 1.53 ± 0.20 | 0.09 ± 0.25 | 0.13 ± 0.30 |  |  |  |
| 16.80 |  | Cy-C17:0 | Bacteria | 0.81 ± 0.20 | 5.18 ± 3.80 | 0.07 ± 0.16 |  |  |  |
| 16.98 | C17:0 | C17:0 | Bacteria | 0.71 ± 0.38 | 0.25 ± 0.66 | 0.14 ± 0.23 |  |  |  |
| 17.44 | C17:0 (?-CH_3_) | Me-C17.0 | Bacteria | 2.66 ± 0.51 |  |  |  |  |  |
| 17.58 | C18:2 (9E,12E) | C18:2ω6 | - | 0.31 ± 0.00 |  | 0.16 ± 0.36 |  |  |  |
| 17.72 | C18:1 (9E/9Z/11) | C18:1 (ω7/9) | - | 5.51 ± 0.65 | 3.00 ± 3.64 | 3.11 ± 1.93 | 3.89 | 3.84 | 59.87 |
| 18.01 | C18:0 | C18:0 | - | 4.29 ± 0.44 | 3.86 ± 3.33 | 11.47 ± 7.03 | 2.82 | 10.12 | 17.69 |
| 18.15 | C19:1 (18-CH_3_) | iC19:1 | Bacteria | 6.18 ± 1.39 |  |  |  |  |  |
| 18.46 | C18:0 (9/10/11-CH_3_) | 9/10/11-Me-C18:0 | Bacteria | 23.98 ± 3.36 | 0.62 ± 1.27 | 10.56 ± 15.93 |  |  |  |
| 18.58 | C19:0 (17-CH_3_) | aiC19:0 | Bacteria | 1.01 ± 0.36 |  |  |  |  |  |
| 18.76 |  | Cy-C19:0 | Bacteria | 1.48 ± 0.59 |  | 6.83 ± 13.53 |  |  |  |
| 19.20 | C20:5 (5,8,11,14,17)C20:4 (5,8,11,14) | C20:5ω3/  C20:4ω6 | Algae |  | 2.89 ± 0.86 |  | 10.15 | 0.25 |  |
| 19.88 | C20:1 (6/11) | C20:1 (ω9/14) | - |  | 0.55 ± 0.50 |  |  |  |  |
| 20.39 |  | C21:1 ω7 | - |  |  | 0.90 ± 1.58 |  |  |  |
| 20.79 | C22:6 (4Z, 7Z, 10Z,13Z,16Z,19Z) | C22:6ω3 | Algae^11^ |  | 6.89 ± 2.32 |  |  |  |  |
| 21.35 | C22:2 (13E, 16E) | C22:2ω6 | - |  |  | 0.18 ± 0.41 |  |  |  |
| 21.58 |  | C22:1 | - |  | 0.86 ± 0.95 |  |  |  |  |
| 23.18 | C24:1 (15) | C24:1ω9 | Sponge |  | 5.12 ± 2.85 | 0.49 ± 0.77 |  |  |  |
| 23.35 | C24:0 | C24:0 | - |  |  |  | 1.67 |  |  |
| 23.64 | C24:1 (24-CH_3_) | iC25:1ω7 | Sponge | 2.01 ± 0.52 | 1.52 ± 1.13 |  |  |  |  |
| 24.04 |  | C25:1 | Sponge |  | 0.74 ± 0.47 |  |  |  |  |
| 24.40 |  | C26:3 | Sponge |  | 13.41 ± 3.57 |  |  |  |  |
| 24.53 | C26:2 (5,9) | C26:2ω17 | Sponge | 1.16 ± 0.66 | 14.47 ± 4.39 |  |  |  |  |
| 24.86 | C26:2 (9,19) | C26:2ω7 | Sponge | 10.14 ± 1.53 | 3.54 ± 1.86 |  |  |  |  |
| 24.92 |  | C26:1 w7/9 |  |  |  | 3.60 ± 1.51 |  |  |  |
| 24.94 | Me-C26:2 (5,9) | Me-C26:2ω17 | Sponge | 1.74 ± 0.93 |  |  |  |  |  |
| 25.09 |  | C27:3 |  |  | 2.30 ± 1.11 |  |  |  |  |
| 25.31 | C27:1 (20) | C27:1ω7 | Sponge |  | 9.53 ± 2.41 |  |  |  |  |
| 25.93 | C28:3 (5,9,23) | C28:3ω5 | Sponge |  | 18.59 ± 7.76 | 6.61 ± 11.70 |  |  |  |
| 26.15 | C28:2 (11,21) | C28:2ω7 | Sponge | 1.43 ± 0.66 | 3.12 ± 2.15 | 1.30 ± 2.04 |  |  |  |
| 26.50 | C29:3 (5,9,22) | C29:3ω7 | Sponge |  | 3.84 ± 1.20 | 0.24 ± 0.53 |  |  |  |
| 26.83 | C29:2 (5,9) | C29:2ω20 | Sponge |  | 2.15 ± 1.79 |  |  |  |  |
| 27.20 | C30:3 (5,9,23) | C30:3ω7 | Sponge |  |  | 48.18 ± 30.86 |  |  |  |

**Supplementary Table S4.**  Results of the pairwise comparisons for the one-factor Monte Carlo PERMANOVAs testing for differences between the deep-sea sponge species *Geodia barretti* (Demosponge, HMA), *Hymedesmia paupertas* (Demosponge, LMA) and *Vazella pourtalesii* (Hexactinellid, LMA),in the contribution of bacterial-specific and sponge-specific PLFAs to the total PLFA profile of each species. df = degrees of freedom, *t* = *t*-statistic, *p*_(MC)_ = Monte Carlo *p* value. Values in bold are statistically significant (*p* < 0.05).

|  |  |  | df | *t* | *p*_(MC)_ | Unique Permutations |
| --- | --- | --- | --- | --- | --- | --- |
|  |  |  |  |  |  |  |
|  | **Bacteria specific PLFAs** | |  |  |  |  |
|  |  | *G. barretti * V. pourtalesii* | 15 | 6.336 | **<0.001** | 5911 |
|  |  | *G. barretti * H. paupertas* | 16 | 23.329 | **<0.001** | 8514 |
|  |  | *V. pourtalesii * H. paupertas* | 11 | 0.980 | 0.346 | 1253 |
|  | **Sponge specific PLFAs** | |  |  |  |  |
|  |  | *G. barretti * V. pourtalesii* | 15 | 4.377 | **<0.001** | 6830 |
|  |  | *G. barretti * H. paupertas* | 16 | 15.644 | **<0.001** | 8490 |
|  |  | *V. pourtalesii * H. paupertas* | 11 | 0.543 | 0.599 | 1711 |

**Supplementary Figure S1. Photographs of the North-Atlantic deep-sea sponge species used in the study.** (**A**) *Geodia barretti* (Demosponge, HMA) (**B**) *Hymedesmia paupertas* (Demosponge, LMA) (**C**) *Vazella pourtalesii* (Hexactinellid, LMA), (courtesy of Lindsay Beazley, DFO).


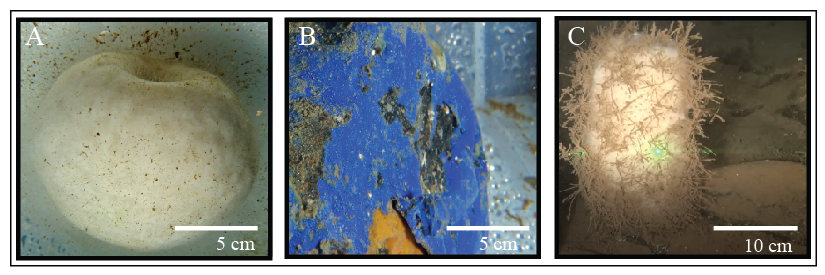


**Supplementary Figure S2. Oxygen profiles for all deep-sea sponge specimens incubated with ^13^C-^15^N enriched food sources**. Dissolved oxygen concentrations (mg L^-1^) in time (min) depicted for (**A**) *Vazella pourtalesii* fed with ^13^C-^15^N-enriched-DOM, **(B)** *Vazella pourtalesii* fed with ^13^C-^15^N-enriched-bacteria, (**C**) *Geodia barretti* fed with ^13^C-^15^N-enriched-DOM, (**D**) *Geodia barretti* fed with ^13^C-^15^N-enriched-bacteria, (**E**) *Hymedesmia paupertas* fed with ^13^C-^15^N-enriched-DOM, (**F**) *Hymedesmia paupertas* fed with ^13^C-^15^N-enriched-bacteria. Dashed red lines indicate the moments of water exchange and addition of a new food pulse.


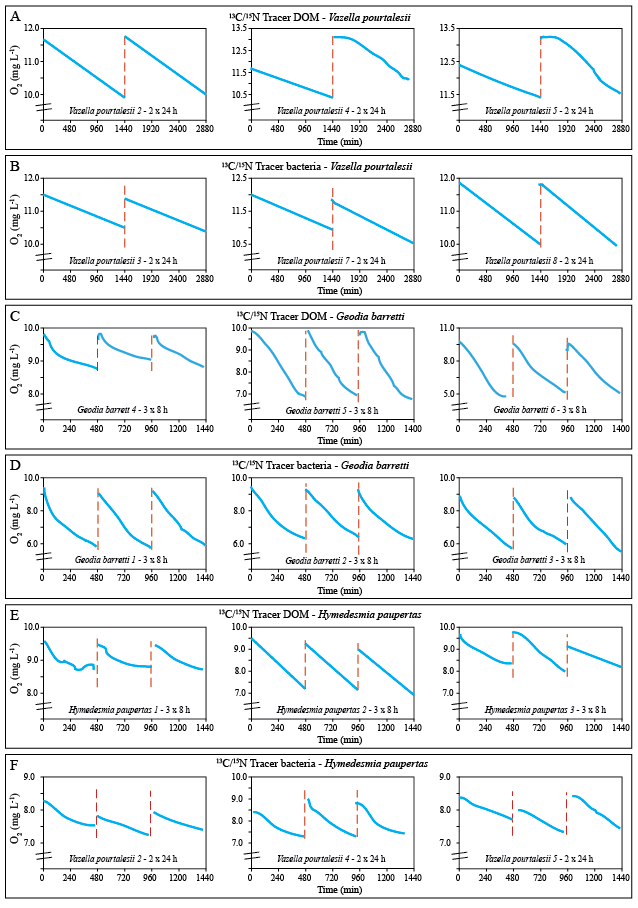


**Supplementary Figure S3. Phospholipid-derived fatty acid (PLFA) profiles of cyanobacterial- and diatom-derived DOM and bacterial substrates**. Values are presented as percentages of the total PLFA content.


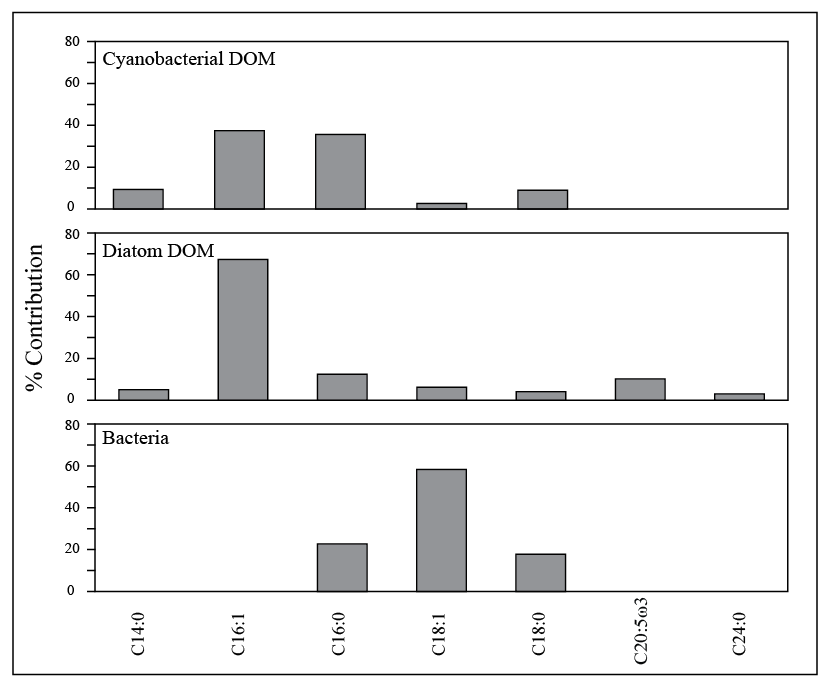


**Supplementary Figure S4. Relative distribution of ^13^C-enriched DOM and bacteria into sponge-specific, bacterial-specific, and unspecific phospholipid-derived fatty acids (PLFAs).** Bacterial-specific (red), sponge-specific (blue), and unspecific PLFAs (grey) are shown (**A)** *Geodia barretti* (DOM: *n* = 3, bacteria: *n* = 4) , (**B)**: *Hymedesmia paupertas* (DOM: *n* = 3, bacteria: *n* = 3), and (**C**) *Vazella pourtalesii* (DOM: *n* = 1, bacteria: *n* = 3). HMA = high microbial abundance, LMA = low microbial abundance.


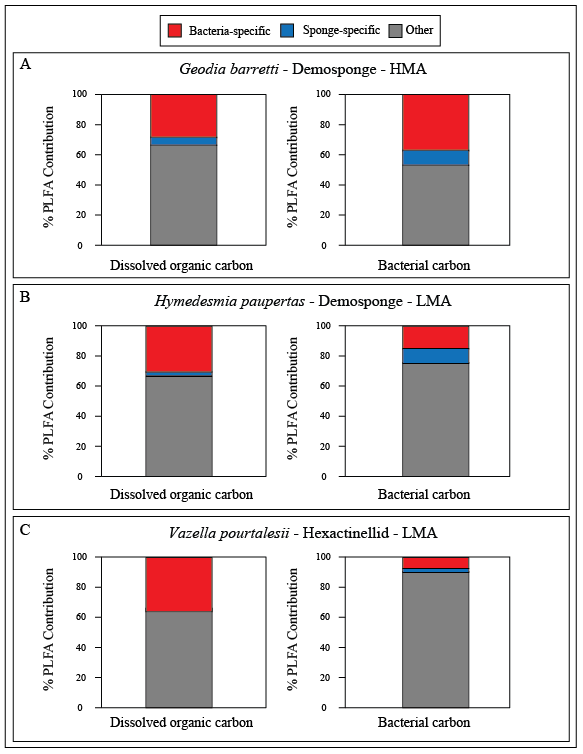

Supplement: Supplementary file 1 — Supplementary Information. [file 41598_2020_74670_MOESM1_ESM.docx]
